# Supplementary material for: Complexome profiling of the Chlamydomonas psb28 mutant reveals TEF5 as an early PSII assembly factor
Source: Plant Cell. 2025 Mar 17;37(6):koaf055. doi: 10.1093/plcell/koaf055 (PMC12164590; doi:10.1093/plcell/koaf055)
Supplement: koaf055_Supplementary_Data [file koaf055_supplementary_data.zip › psb28_interactive.zip!/plots/intensity/qualityPlots/peptides/NUOA8_Cre07.g333900.t1.1.html]

### Cre07.g333900.t1.1

Trivial name: NUOA8  
SeqCoverage [median]: 24.50  
Mass: 12.52 kDa  
  
 

| Sequence | MissedCleavage | Mass [Da] | wtIntensity | muIntensity | Score |
| --- | --- | --- | --- | --- | --- |
| DKQTAFEK | 1 | 965.48 | 0.00 | 1767.12 | 150.36 |
| QTAFEK | 0 | 722.36 | 388.59 | 556.52 | 124.34 |
| TSNTVR | 0 | 676.35 | 1035.92 | 0.00 | 92.69 |
| VPYSHELYAMSR | 0 | 1451.69 | 21356.20 | 21660.22 | 101.60 |
|  |  |  | 22780.7 | 23983.9 |  |
